# EssOilDB: a database of essential oils reflecting terpene composition and variability in the plant kingdom

**DOI:** 10.1093/database/bau120

**Published:** 2014-12-20

**Authors:** Sangita Kumari, Sachin Pundhir, Piyush Priya, Ganga Jeena, Ankita Punetha, Konika Chawla, Zohra Firdos Jafaree, Subhasish Mondal, Gitanjali Yadav

**Affiliations:** Computational Biology Laboratory, National Institute of Plant Genome Research (NIPGR), New Delhi 110067 India

## Abstract

Plant essential oils are complex mixtures of volatile organic compounds, which play indispensable roles in the environment, for the plant itself, as well as for humans. The potential biological information stored in essential oil composition data can provide an insight into the silent language of plants, and the roles of these chemical emissions in defense, communication and pollinator attraction. In order to decipher volatile profile patterns from a global perspective, we have developed the ESSential OIL DataBase (EssOilDB), a continually updated, freely available electronic database designed to provide knowledge resource for plant essential oils, that enables one to address a multitude of queries on volatile profiles of native, invasive, normal or stressed plants, across taxonomic clades, geographical locations and several other biotic and abiotic influences. To our knowledge, EssOilDB is the only database in the public domain providing an opportunity for context based scientific research on volatile patterns in plants. EssOilDB presently contains 123 041 essential oil records spanning a century of published reports on volatile profiles, with data from 92 plant taxonomic families, spread across diverse geographical locations all over the globe. We hope that this huge repository of VOCs will facilitate unraveling of the true significance of volatiles in plants, along with creating potential avenues for industrial applications of essential oils. We also illustrate the use of this database in terpene biology and show how EssOilDB can be used to complement data from computational genomics to gain insights into the diversity and variability of terpenoids in the plant kingdom. EssOilDB would serve as a valuable information resource, for students and researchers in plant biology, in the design and discovery of new odor profiles, as well as for entrepreneurs—the potential for generating consumer specific scents being one of the most attractive and interesting topics in the cosmetic industry.

**Database URL**: http://nipgr.res.in/Essoildb/

## Introduction

Plants are capable of generating a diverse spectrum of volatile organic compounds (VOCs or essential oils, also known as ethereal oils) for maintaining communication and fruitful adaptive interaction with their environment. Extensive research on plant volatile emission has shown these to be important protective and signaling molecules under oxidative, thermal and pathogen stress and can act both as allelochemical and neighbor detection signals, thus constituting a platform for plant–plant interaction (1, 2). Herbivore-challenged plants are known to emit volatiles not only to invite the natural enemies of herbivores (parasitoids and predators), but also to aid neighboring plants by inducing defense response in them (3–5). Along side of such roles in defense, VOCs have enormous applications in pharmaceutical, industrial and agribiotechnological areas. Since antiquity, plant-originated natural products have been applied for their medicinal and organoleptic properties. They are extensively used in food and cosmetics industry for generating flavor and fragrances due to their pleasant scent (6). The significant role of volatiles in the prevention and treatment of human diseases is attributed to their specific activities against microbes, cancer, cardiovascular problems and diabetes (7–10). Besides these properties, they have also shown to be associated with hepatoprotective, spasmolytic and carminative activities (11, 12). Recent reports on the analysis of their free radical scavenging capacity have suggested that they can act as promising natural antioxidants as well (13–15).

The essential oils (where the term ‘essential’ denotes that the oil has the fragrant essence of the source plant) are multicomponent chemical combinations having terpenes as their major constituents along with other non-terpene compounds (16), and can be obtained from plants with the help of an appropriate extraction method (17). The traditional extraction methods like hydrodistillation, steam distillation, solvent extraction and expression under pressure, are more common although advanced techniques such as supercritical fluid and subcritical water extraction have been used in recent times (16, 18). Interestingly, several independent studies have demonstrated that the composition of essential oils may depend upon various taxonomic, geomorphologic, environmental and genetic factors such as population variation, physiological age, seasonal change, specific plant organ and stress conditions (19–23). However, the association between volatile profiles with various factors and exact mechanism behind their significant role in several stress responses are poorly understood. The systematic compilation of essential oil profiles along with the additional information about a variety of biotic and abiotic factors may provide insights for understanding the roles of volatiles in the life of a plant.

Although a few commercial and public-domain resources exist that contribute to this area, these are mainly dedicated to aromatherapy and the perfumery industry and thus have limited scope or applicability in rational scientific research. As a result, there is currently no essential oil database in the public or commercial arena that provides comprehensive information on essential oil composition data and related geo-morphological factors at the time of collection and extraction. This lacuna has been addressed by this work, wherein we present the ESSential OIL DataBase (EssOilDB), a continually updated knowledge resource for plant essential oils containing experimentally recorded essential oil profiles from several published reports. For each record in the database, the scientific name and plant family information of the source plant, origin of the plant material and identification method used in the analysis is provided along with complete literature citation. Additional factors such as prevailing stress, environmental conditions, invasive nature of plants etc have also been taken into account while compiling the data. For each compound in EssOilDB, chemical formula, CAS number, classification, reported biological activity and percentage composition are also provided. In addition, EssOilDB returns preliminary statistical analyses on user queries. EssOilDB (version 1.0) facilitates various functions such as data search, data download, data statistics along with pie charts that can assist users in finding, retrieval and analysis of data.

In order to emphasize the use of EssOilDB in plant biology, we also highlight the natural diversity of plant isoprenoids through an in-depth analysis of records in this database. Isoprenoids represent one of the most ancient and widespread classes of structurally and functionally rich biomolecules known to man. The plant kingdom exhibits tremendous variation in their chemistry and roles, ranging from primary metabolism to secondary metabolism and specialized ecological interactions with the environment, and a majority of terpenoids, specially sesquiterpenes and monoterpene derivatives are released by plants in the form of volatile emissions. Isoprenoids are derived from the universal C5 precursor isoprene, and terpene synthase (TPS) enzymes are key players in the generation of isoprenoid diversity, catalyzing one of the most complex reactions known to chemistry and biology. Infact, TPSs can generate such a tremendous diversity of compounds using the regiochemical and stereochemical intricacy of their cyclization reactions that the complete terpenoid chemical library of a given plant has been termed as the ‘terpenome’ (24, 25). The ongoing genomic revolution has provided an unprecedented opportunity for genome mining efforts toward discovery and characterization of the plant terpenome.

In the following sections, we present a summary of the contents of EssOilDB, its availability and limitations, and a comparison with other available databases that have served as a benchmark for us. Thereafter we present specific examples to illustrate the use of this database in stress biology, terpene diversity and variability in the plant kingdom. We also highlight the applicability of EssOilDB and its ability to complement high throughput data in the current era of comparative genomics.

## Methodology

### Data collection rationale

Essential oil profiles were collected from literature focusing on specialized peer reviewed journals like the Journal of essential oil research (JEOR), Journal of agricultural and food chemistry, Flavour and fragrance journal, Fitoterapia, Phytochemistry, the Journal of food processing & technology. The database includes records from more than 40 scientific journals, both national and international, some of which are continent- and nation-specific such as the African journal of Biotechnology, Croatica Chemica Acta, Brazilian Journal of Pharmacognosy, Macedonian Journal of Chemistry and Chemical Engineering and the Proceedings of the Royal Society of New South Wales, among others. One of the major features of EssOilDB is that we take into account a number of bio-geological conditions that influence essential oil compositions, such as environmental and physiological circumstances, plant parts used for oil extraction, invasiveness and geographical distribution etc. The database has been envisioned to enable users to combine these parameters in a single search and through this get an insight into how these factors individually or in concert influence volatile profiles of the species in question. Towards this, we are constantly adding new articles with the objective of improving and maintaining the diversity and accuracy of data in EssOilDB. The rate of data addition usually depends upon the quality and quantity of information in a given article, average rate of data addition varying from few articles per day to one article in a few days, preceded always by a thorough manual study of papers. It is critical to emphasize that it is not our aim to randomly incorporate every single essential oil article that may ever get published. Rather we hope to create an intelligent biological resource, representing the diversity of volatiles in the plant kingdom or representing multiple essential oil profiles corresponding to different experimental conditions. For a plant that is already in the database, we avoid redundancy by adding more data from the same geographical region, unless it represents a distinct divergence from the existing information. The identity of plant species, family, source plant organ, geographical location, time of collection, environmental condition is extracted from each record and incorporated as a key into the database as explained in the next section. For each compound in the records, information is collated for CAS number, chemical formula, amount of volatile recorded. The database also links the identification method with each record and the major identification techniques include gas chromatography, gas chromatography/mass spectrometry, gas chromatography/flame ionization detector and gas chromatography with electron impact mass spectrometry. Evidence for invasive nature of a given plant under study has been incorporated wherever available, along with whether or not there were any adverse/stress conditions at the time of harvest that may have a bearing on the essential oil profiles. The information about invasive nature of source plant in specific geographical location has also been incorporated, wherever available in these records, and also from a variety of online databases such as global invasive species database (http://www.issg.org/data base/welcome/), the database of introduced, invasive and noxious plants (http://plants.usda.gov/java/noxiousDriver) and the invasive database (www.invasive.org/)*.* Information about biological activity and general chemical classification of the compounds was extracted from peer reviewed journals or manually curated from information provided in public websites available on the internet such as Dr. Duke's Phytochemical and Ethnobotanical Databases available at ‘http://huilesutiles.eu/docs/niaouli.pdf’. In order to overcome the issue of chemical synonyms of a given compound, we have provided IUPAC name of compounds. Furthermore, each compound in EssOilDB is linked to pubchem (https://pubchem.ncbi.nlm.nih.gov). Apart from seamless cross-platform compatibility, this cross-platform link provides access to detailed chemical information provided by pubchem including IUPAC name, formula, classification, structure and multiple synonyms. Benchmarking of EssOilDB was done by performing a feature-by-feature comparative assessment with existing public and commercial databases that are considered significant for contributing information on essential oils, such as the EOUdb (Essential Oil University database), AromaWeb (http://www.aromaweb.com/essentialoils/), the Cropwatch files (http://www.cropwatch.org/) and the Phytochemical and Ethnobotanical Databases (http://www.ars-grin.gov/duke/).

### Data extraction and query interface

**Figure 1.**
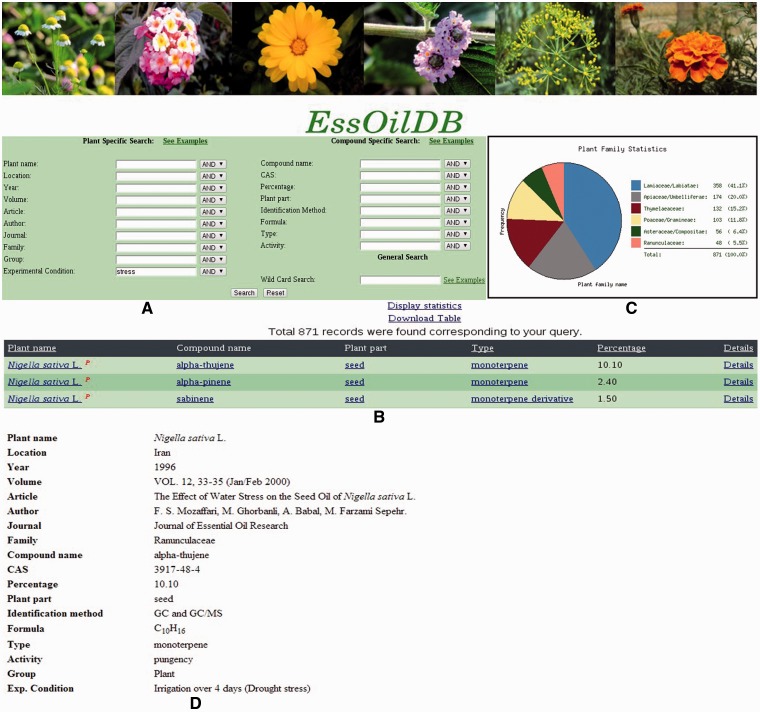
Snapshots of query submission in EssOilDB using ‘Stress’ as query in the Experimental condition key. (**A**) Main search page with various plant specific and compound specific keys. (**B**) Tabular output for all the matched records and links to (**C**) statistics of query in the entire database and (**D**) Details with more information of each record including citation, chemical structure, biological activity and other features.

EssOilDB has been developed using MySQL5.1.52, Apache, PHP and PERL and currently runs on a red hat server. Emission tables from published data are pasted directly into SQL formats using simple shell scripts. Additional properties of emitted compounds such as formula, chemical classification, CAS number and biological activity are also incorporated through bio-perl scripts. Data extraction is partially automated since the majority of articles published in the past few decades are available online in HTML formats and their volatile profiles do not require an OCR scan. However, older articles (some EssOilDB records date back a century) are available as scanned images that require manual entry, or an OCR scan followed by manual checking. For each article incorporated, two MySQL tables are created with file handles to the source plant species and chemical compound respectively. Each record in the database corresponds to the amount of emission of a particular compound in a specific oil profile. All EssOilDB records, whether old or new, are checked at least twice, since we add biological/chemical information over and above the data available in the article alone. This ensures error minimization since each new emission table data has to go through several rounds of data matching from other resources. Figure 1 shows the main query interface of EssOilDB. The interface is user-friendly and records can be searched by wild cards as well as various plant and compound specific keys. In all there are ten plant specific keys and eight compound specific keys, which is also the highest number of searchable keys in any existing essential oil database. The major plant specific keys are species name, plant family, geographical location, environmental condition and citation whereas compound specific keys include name, CAS number, compound category, identification method, source plant material and biological activity. The search result includes the total number of records found matching the query along with a list of matching records with general information like plant name, compound name, compound category/type, source material, percentage emission and a link for each record providing citation details. On clicking the details link, one can access extensive information about source plant, extracted compound and citation for each record. All output data can be downloaded in tab delimited text format. Statistics pertaining to each query can be visualized and downloaded in image format via specific pattern display links provided on each result page, as shown in Figure 1.

## Results

### Database content

**Figure 2.**
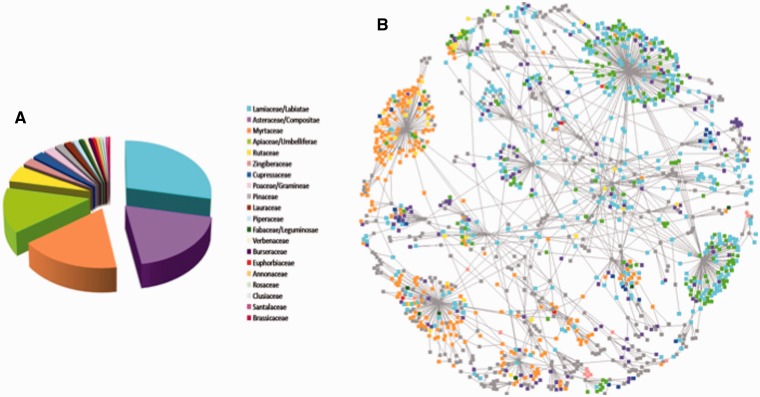
Summary representation of plant records in the EssoilDB. (**A**) The most populated families in the database, depicting Lamiaceae (cyan) and Asteraceae (pink) to have maximum number of records in the database, followed by Myrtaceae (orange) and Apiacae (green). (**B**) A network representation of the geographical distribution of these families, showing the connected component of the plant data network. Peripheral nodes represent plant species and hub nodes represent geographical locations. Note that Color codes are maintained between the panels.

As described in methods, each EssOilDB record corresponds to the amount of emission of a particular compound in a specific oil profile. Further, in case a single journal article lists three different sets of volatile profiles, say for three different plant parts, or under three independent stresses, we treat the datasets as three independent records. Currently, the database contains a total of 123 041 such records spanning a century of published reports of essential oil profiles, starting from early 1900s to date. These records have been sourced from over 1520 citations and the data includes 1618 plant species, subspecies or varieties representing 92 distinct taxonomic families encompassing the entire range from ancient and lower plants like chlorophytes and mosses, to the gymnosperms and angiosperms. Figure 2 provides an estimate of the geographical and taxonomical distribution of the records and it can be seen that families over represented in the data belong to high volatile content taxa such as the Myrtaceae, Apiaceae and Lamiaceae. The extensive amount of data for these taxa, along with lists of essential oils from mosses and volatile hydrocarbons from algae to pines may assist in deducing patterns between phylogeny of plants and their emission profiles. Essential oils were extracted from distinct aerial or underground plant structures such as leaf, root, rhizome, seed, bark, flower, fruit or any combination of these or even the whole plant (17). The material used for extraction may further be fresh, dry, juice extract or lab-dried, or it may have been harvested at a certain developmental stage such as ripe, semiripe or unripe fruits, woody or non-woody branches etc. EssOilDB integrates these aspects wherever possible in order to collate available information about the material used for obtaining essential oils and users can browse through more than 160 different types of plant part/sub-part categories. For each compound, EssOilDB includes known information about any associated biological activity, and the current version of the database lists these properties for 44 787 records. These records comprise various combinations of over 200 distinct activities that have pharmacological, commercial or agroindustrial relevance. In view of the fact that a majority of essential oils comprised terpenoid compounds and their derivatives, the database contains information about the type or general chemical classification, wherever possible, such as a the specific class of terpenes. For example, alpha-pinene is a monoterpene, while gamma-murolene belongs to the sesquiterpene category. The database contains relatively fewer diterpenes records; namely abietol, cembrene, isopimara-7,15-diene, phytane, phytol and sclareol, which in turn, reflect the fact that diterpenes are generally not volatile in nature. Currently more than 40 categories are recorded in this section, providing information on whether a compound under consideration is an alcohol, fatty acid, ketone, ether, mono-, di- or sesqui-terpene etc. As mentioned earlier, evidence for invasive nature of a given plant under study has been incorporated wherever available, and EssOilDB presently has 2806 essential oil records obtained from invasive plants located across the major continents of the world. The data is specially relevant in the Indian context as it contains volatile profiles of highly invasive species in various parts of India, of which *Lantana camara* has the highest number of records followed by *Ageratum conyzoides*. The analysis of these essential oil profiles of invasive plants may provide the opportunity to identify the specific patterns present between plant volatiles and their invasive nature if any. Further, this knowledge may also be used to find the significance of specific volatiles in the development of invasive nature and these concepts may be introduced practically to gain benefit in agricultural perspectives.

### Benchmarking analysis

We have benchmarked EssOilDB by carrying out a comparative assessment of its features with existing public databases contributing significant information on essential oils. Some of these are EOUdb (Essential Oil University database), AromaWeb (http://www.aromaweb.com/), Phytochemical and Ethnobotanical Databases (http://www.ars-grin.gov/duke/) and the Cropwatch files (http://www.cropwatch.org/). EOUdb is an online chemical reference database (http://essentialoils.org/eoudb/) with a login requirement, and it provides information on essential oils and their chemical composition, although details about source plant, taxonomy or ecological factors is entirely lacking. Aromaweb is dedicated to holistic aromatherapy and accordingly limited to just over one hundred essential oil profiles that are most important for the perfumery industry. The Phytochemical and Ethnobotanical Databases are not limited to essential oils and contain vast information on all kinds of phytochemicals including sugars and proteins as well. There is also no direct way to browse the data, users have to necessarily type the name of the chemical, plant or activity of interest. Since this resource is motivated by diagnosis and medication, the records do not mention any citation details or extraction methods, providing only a list of chemicals along with activities, benefits and source plant organs and references to personal notes or text books. The Cropwatch files essentially represent a resource for surveys and critical opinions about aromatic products from commercial sources, rather than an essential oil database, per se. Many commercial essential oil databases such as Essential Oil Bioactivity Research Database, Essential oil component database (http://www.quintessential.uk.com/index.php), LabAroma (http://www.labaroma.com) are also available, but they emphasize on literature and information based on the bioactivity aspect of essential oils, catering mainly to the cosmetic and perfumery industry. In addition to this, ESO 2000 (http://www.leffingwell.com/baciseso.htm) for quantitative analysis of essential oil is also commercially available but it has no contextual data on stress or invasive nature of the plants under study. A detailed comparative chart listing the features available in various databases is provided in See online supplementary material for Supplementary Table S1. It clearly reveals EssOilDB as the only public database that is dedicated to the biology of essential oils and may reliably be used for scientific research on patterns and profiles of essential oils and their variations across a variety of contextual situations.

### Association of essential oil data with genetic, phenological and ecological factors

**Figure 3.**
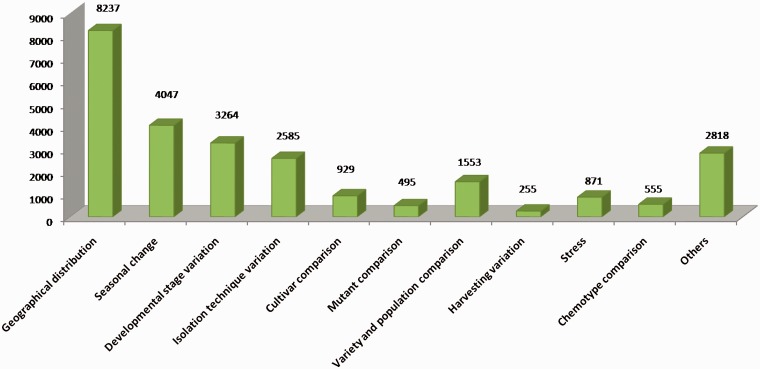
Depiction of the various kinds of experimental conditions in EssOilDB and their corresponding number of records.

**Figure 4.**
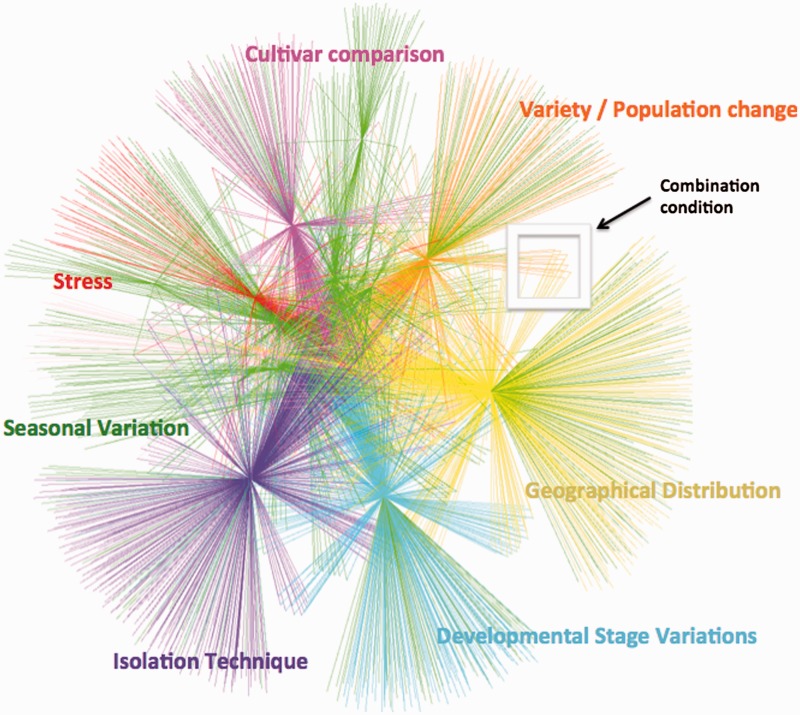
Network representation of the compound data in EssOilDB. The view summarizes essential oils emitted under some of the variable conditions depicted in Figure 3, each condition being depicted in distinct colors. Edges are colored by the specific variable condition being studied. Peripheral nodes represent compounds while hub nodes represent a specific feature of the data, as annotated. Nodes towards the periphery of the network are unique to a given condition, whereas as one moves from the periphery to the center, nodes represent compounds that are common to an increasing number of conditions. For example, the inset shows the nodes (compounds) that are common to two distinct features, namely, geographical location variations (yellow) and population based variations (orange). Note that a large number of compounds synthesized under stress (red edges) are unique, whereas compounds that are released based on developmental stage variations or seasonal variations are common to a majority of the conditions analyzed.

Several studies have been carried out to compare essential oil profiles under different experimental conditions. EssOilDB offers a major advancement to such studies as it provides information about specific environmental factors associated with essential oil records, thereby facilitating a comparative analysis under normal and user-specific plant conditions, which may be studied individually or in combination with other factors. Apart from available volatile profiles under normal conditions, EssOilDB has 30 883 records in which essential oil profiles have been measured and published based on a given factor as depicted in the form of a chart in Figure 3. These records represent variations in developmental stages, spatial distribution, seasonal variation, mutant analysis, isolation techniques, population- or cultivar-based variation, as well as different kinds of abiotic and biotic stress conditions (Figure 3). Geographical distribution studies are the topmost among all factors in the database with 8685 records. The major reports of this section include the analysis of essential oil composition of a plant species across different altitudes, habitats and populations, providing insights about spatial location based intraspecific variations. The second highest factor incorporated in the database is climatic changes (4657) in which the effect of different temperature, rainfall and day length is mapped to the volatile composition profile of a given plant. The transition from one developmental stage to another such as vegetative to reproductive is inter-connected with variety of factors such as photoperiod, temperature, nutrients and hormones. The database has 3393 records in which changes in chemical profiles are studied under various stages of growth and development during the life history of plants. Interestingly, there have been several reports on the combinatorial effect of seasonal change and developmental stage variation on plant essential oil composition, and EssOilDB contains many such records that can be browsed by users. Figure 4 clarifies this aspect with a clustered view of the essential oils emitted under some of the specific conditions described above, each condition being depicted in distinct colors. EssOilDB thereby enables analysis of compounds emitted under user-specific combinations of conditions, such as the oils that may be specific to a given cultivar at a given geographical location. As can be seen from Figure 4, emissions can be distinguished based on whether they are completely unique to a particular feature or may be commonly released between multiple conditions. For example, a large number of compounds synthesized under stress are unique, whereas compounds that are released based on developmental stage variations or seasonal variations are common to a majority of the conditions analysed. Such common compounds may vary by the exact amounts emitted by the respective plants, which may in turn, depend upon the condition or feature being studied. One such example has been provided in the next section, where a plant produces the same compounds before and after stress, but there is a drastic change in the amounts released upon infection. As discussed earlier, there are many isolation techniques used for the extraction of essential oils and the comparative analysis of these techniques are also reported on the basis of quality and quantity of essential oils obtained by applying them. EssOilDB includes 2872 records providing comparative lists of volatiles extracted through different methods. The chemistry of volatiles has been shown to be differential under the effect of genetic variants such as mutants, cultivars, and populations. The database incorporates genetic factor based compositional profiles that may be used for associating genotype variations with the manifestation of distinct chemical phenotypes. Plants exhibit significant variation in essential oil profiles based on variation in ecological factors, such as the nature of habitat, their abundance and abiotic and biotic factors (26). One of the major strengths of EssOilDB is that it can facilitate visualization of emission related variations in diverse plant conditions, providing insights into the cause of such variations. Apart from conditional variations associated with either the plant or the extraction procedure as described above, it can be very valuable to understand compositional variations in the emission itself, and such studies are facilitated in EssOilDB through incorporation of detailed information on the identified chemical compounds within essential oil bouquets, including their registry number, percentage emission, general chemical classification, chemical formula and biological activity, as described in methods.

### Terpene variability in the plant kingdom

**Figure 5.**
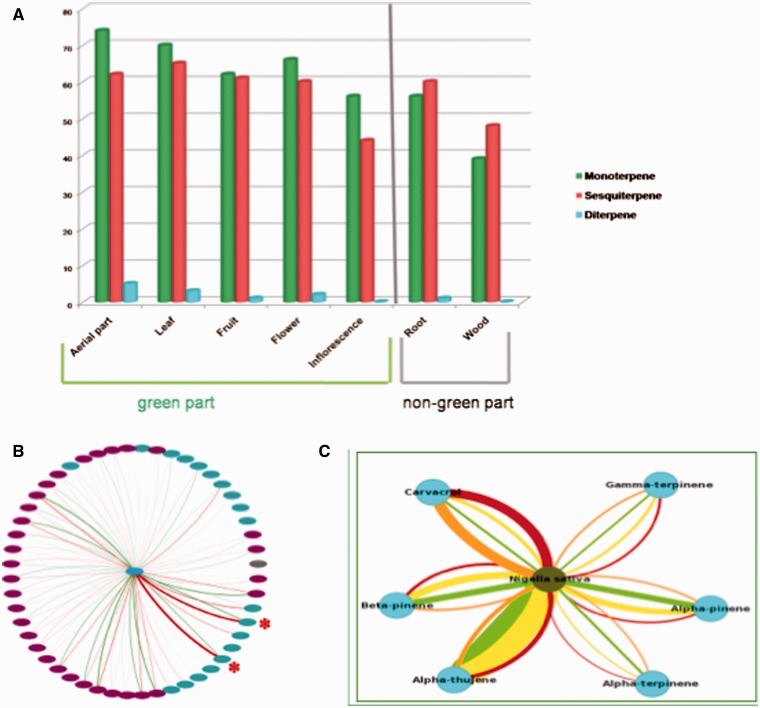
Biological analysis of EssOilDB data for insights into terpene diversity and variability. (**A**) Graphical overview of different type of terpenes (monoterpene, sesquiterpene and diterpene) and their source plant parts. Note that essential oils obtained from root and wood have higher number of unique sesquiterpenes as compared to monoterpenes whereas other plant parts have shown its reverse, reflecting differential activation of plastidial MEP pathway as discussed in the text. (**B**) Terpene emission patterns of *Echinacea purpurea* under normal and biotic (cucumber mosaic virus) stress. Nodes represent compounds and edges represents normal (green) or stressed (red) conditions. Pink nodes represent sesquiterpenes while green nodes represent monoterpenes. Edge width represents percentage emission and it is clearly visible that Myrcene and alpha-terpene emissions (red asterisks) increase under stress. (**C**) Terpene profile network of *Nigella sativa* under normal and drought stress. Green edges represent amount of irrigation over four days. Carvacrol emission increases under drought stress (thick red and orange edges).

As an illustrative example, we have used EssOilDB to carry out an assessment of the relationship between terpene biosynthetic pathways and actual emission records. The biosynthesis of monoterpenes and diterpenes is conventionally believed to be compartmentalized in plastidial territory of the plant where the 2C-Methyl-D-erythritol 4-phosphate (MEP) pathway is known to be located. In contrast, sesquiterpenes and triterpenes are supposed to be synthesized in cytosol through classical mevalonic acid (MVA) pathway (27). This assumption is further supported by recent studies on promoter analysis in *Arabidopsis thaliana* that suggest an abundance of light and circadian clock related motifs in MEP pathway gene promoters reflecting higher expression of this pathway in green tissues as compared to MVA pathway (28). Spatiotemporal expression analysis of genes encoding farnesyl pyrophosphate synthase (FPPS), geranyl pyrophospate synthase (GPPS) and geranylgeranyl pyrophosphate synthase (GGPPS) in *A. thaliana* have also supported the fact that photosynthetic tissues have higher expression of *AtGPPS* and *AtGGPPS* genes*;* generally assumed to synthesize monoterpenes and diterpenes respectively. In contrast, the *AtFPPS* gene*,* which is responsible for the biosynthesis of sesquiterpenes, showed higher activity in roots and seeds as compared to above ground, green parts (28). We used EssOilDB to test this hypothesis and found that green parts of plants (that are likely to have a more active plastidial MEP pathway) do indeed show relatively greater amounts of released hemi, mono and diterpenes, as compared to sesquiterpenes. In contrast, the non-green plant parts, such as the underground regions and woody parts (signifying an absence of plastidial units and therefore a less active MEP pathway) release higher amounts of sesquiterpenes as compared to monoterpenes. This has been depicted in Figure 5A, with emission data records from green (plastidial) parts, leaves, fruits and flowers releasing a higher percentage of monoterpenes as compared to sesquiterpenes, whereas roots and bark or woody parts release more sesquiterpenes, thus significantly adding value to the hypothesis.

### Terpene diversity in the plant kingdom

OilDB data was used in conjunction with genomic data in order to understand how a plant creates the so-called final terpenome, specific to itself, and whether or not plants tap the complete potential for terpene biosynthesis at their disposal according to their genomes. For selected plant species with publicly available genome sequence data, we correlated the ‘actual/observed’ terpene emissions (as recorded in EssOilDB) with ‘expected’ terpene content, (as predicted by a computational identification of TPS genes) for that genome. Table 1 shows this data for 11 distinct plant species, where the terpene potential of the genome (terpenome) has been compared with the observed or actual terpene emission from EssOilDb records. As can be seen from this table, the available emission profiles appear to only partially capture the full terpene potential. For example, in case of *Eucalyptus*, the emission of only 21 monoterpenes have been recorded in literature, whereas the genome of this plant contains genes that code for more than double this number of monoterpene synthases. This is the case with many other plants analysed in this effort, and it may be explained by limitations of record compilations or the unavailability of data under stress for the plant, since these are secondary metabolites induced for specific functions and a large number of biosynthetic genes may lie dormant under natural conditions. Interestingly, a few plants like the legume crop chickpea (*Cicer arietinum*) show a contrasting trend, wherein more terpene emissions have been recorded in literature than the predicted genomic potential (Marked in bold font in Table 1). This means that the number of TPS gene models in the plant genome is less than the total number of terpenes released by that plant, reflecting in turn, the unique ability of TPS enzymes to synthesize multiple products from a single substrate. A single predicted TPS gene product can thus generate many terpene compounds rather than only one, and this ability to create complex odor profiles from a minimal gene set can lead to an expansion of the terpene potential of the respective genome. Further characterization and detailed analysis of such genes may provide insights into the unique molecular mechanism of TPSs that remains poorly understood. This study illustrates the use of EssOilDB for addressing complex and interesting questions in terpene biology and in identification of new avenues for scientific research.

**Table 1.** Observed versus predicted terpenome for various plants

Name of plant	Monoterpene O/P	Sesquiterpene O/P	Diterpene O/P	Total O/P
*Arabidopsis thaliana*	15/18	7/30	0/4	22/52
*Citrus sinensis*	30/34	19/57	0/6	49/97
*Eucalyptus grandis*	21/51	12/65	0/8	33/124
*Ricinus communis*	5/26	0/23	0/10	5/59
*Vitis vinifera*	4/24	12/74	0/10	16/108
*Fragaeria vesca*	**25/11**	6/41	1/12	32/64
*Malus domestica*	3/26	1/57	1/25	5/108
*Citrus clementina*	**21/8**	7/7	0/5	28/20
*Carica papaya*	12/13	1/21	0/9	13/43
*Gossypium raimondii*	13/31	3/68	0/12	16/111
*Cicer arietinum*	**15/7**	0/13	0/4	15/24

A/P: Actual Terpene Emission Records/Predicted No. of TPS genes. Bold values depict contrasting trends, as described in text.

### Alteration in terpene emission profiles under stress

Extensive research has been carried out to elucidate the stress responses of plants, including the investigation of essential oil compositions under adversity. Accordingly, EssOilDB contains records displaying the observed variability of volatile compositions in different abiotic and biotic stress conditions; and this data would be indispensable in mapping volatile release to the extent of stress. Emission profiles of plants often show significant changes in oil composition under various biotic and abiotic stress conditions. For example, EssOilDb records show that viral stress induces the release of two monoterpenes *Echinacea purpurea* (29)*.* As depicted in Figure 5B, *E.purpurea* releases over 50 compounds during cucumber mosaic virus infection, and although these compounds are also released under normal conditions, the percentage emissions vary. Thus, it can be clearly seen that myrcene and alpha-pinene (both monoterpenes) are synthesized and released in higher amounts under stress. Similarly, sesquiterpenes emission has been recorded to increase under blue light in *Ocimum selloi*, and as shown in Figure 5C, drought stress records from EssOilDB reveal the induction of monoterpene carvacrol in *Nigella sativa* whereas other monoterpenes show decreased emission under stress (30, 31). The database also contains records exposing the induced emission of beta-caryophyllene (a sesquiterpene) in severe salt stress from stems alone, suggesting that emission profiles can significantly vary with plant parts. Interestingly, emission patterns recorded in EssOilDB show significant difference in cases of natural and artificial infection, or based on the noxious state of a plant, or whether it is an introduced plant. This complex emission pattern indicates that the specific isoprenoid compound, type and degree of stress conditions, plant species and plant parts are dynamically co-ordinated by a multi-layered and finely tuned regulatory network that excellently controls isoprenoid biosynthesis and its emission.

## Conclusion and future directions

As described earlier, essential oils have huge potential in pharmacology both as preventive and treatment agents for a range of health disorders. Further, they have also shown to be involved in aromatherapy and facilitating skin penetration and used for transdermal delivery of medicines (16). In addition to therapeutics, their commercial value in food and cosmetic industry has also increased tremendously. Creation of EssOilDB is an attempt to provide a systematic compilation of essential oil profiles along with the details of their sources for the benefit of not only scientific community but also for the layman, entrepreneurs and farmers, interested in exploring volatiles and their properties. As evident from the benchmarking analysis presented here, EssOilDB is the first and only database that enables a rigorous scientific assessment of plant essential oils in context of their surroundings and in a comparative manner. It takes into account the influence on essential oil composition of a number of environmental and physiological conditions, plant parts, invasiveness and geographical distribution. The database makes it possible to combine these parameters in a single search and through this get an insight into how these factors individually or in concert influence the essential oil composition of the plant in question. It is a freely available user-friendly database, which will support both computational biologists as well as experimental researchers investigating the changing vocabulary of essential oils with various influences, and applications thereof. It must be emphasized here that although we carry out cross-platform checks and multi-source record integration, data in EssOilDB is essentially no better than the input sources and these may be of very variable reliability especially concerning identification of essential oil compounds. The most common way of ‘identifying’ compounds is to compare an obtained mass spectrum with those in a mass spectral database. However, a compound is only properly identified if it is compared with an authentic standard. For example, very few sesquiterpenes are available commercially and therefore often only identified through mass spectral databases often leading to ironic results. However, our method of working at the level of a whole class of compounds such as sesquiterpenes, monoterpenes or diterpenes partly eliminates this problem. We hope to expand EssOilDB in future versions, with increased dataset of specific environmental conditions and comprehensive details of essential oils, including information on potential industrial applications of compounds, their chemical structure, biosynthetic sources and genomic properties, if any. Regular literature updates will be carried out from upcoming reports of essential oil composition analyses. We are also working on a blueprint to incorporate data analysis tools using graph theoretical approaches in order to provide better insights into user-specific searches. Implementation of novel ideas and specific requirements suggested by users will be attempted and technical feedback is always welcome.

## Supplementary Material

Supplementary Data
