# Supplementary material for: EssOilDB: a database of essential oils reflecting terpene composition and variability in the plant kingdom
Source: Database (Oxford). 2014 Dec 20;2014:bau120. doi: 10.1093/database/bau120 (PMC4273207; doi:10.1093/database/bau120)
Supplement: Supplementary Data [file supp_2014_bau120_index.html]

EssOilDB: a database of essential oils reflecting terpene composition and variability in the plant kingdom — Supplementary Data 

# EssOilDB: a database of essential oils reflecting terpene composition and variability in the plant kingdom

## Supplementary Data

files

**Files in this Data Supplement:**

- Supplementary Data - docx file
