# Supplementary material for: EssOilDB: a database of essential oils reflecting terpene composition and variability in the plant kingdom
Source: Database (Oxford). 2014 Dec 20;2014:bau120. doi: 10.1093/database/bau120 (PMC4273207; doi:10.1093/database/bau120)
Supplement: Supplementary Data [file supp_bau120_Supplementary_Data.docx]

Supplementary Data for Kumari et al

This data contains Supplementary Table 1: A feature by feature comparative analysis of various public databases relating to Essential Oils. It is clear from this data that EssOilDB provides more information than any of the existing resources in multiple aspects.

| **Feature** | **EssOilDB** | **EOUdb** | **AromaWeb** | **Phytochemical and Ethnobotanical Databases** | **The Cropwatch Files** |
| --- | --- | --- | --- | --- | --- |
| **Oil composition Break up** | Provided with amounts | Provided with amounts | Provided without amount | Not provided | Provided with amounts in few cases |
| **No. of Plant Species** | 1528 | 826 | 107 | 2376 | About 20 species |
| **Taxonomic families** | 78 | 97 | Not Provided | Provided | Not Provided |
| **Origin of Data** | Global | Global | Not Provided | Not provided | Provided |
| **No. of Essential Oils** | 2868 | 2571 | 110 | Not provided specifically (focused on chemicals rather than oils) | Not specifically provided, main focus is on opinion |
| **Citation Details** | Provided | Provided | Provided | Provided (mostly to personal notes) | Provided |
| **Statistical Analysis** | Enabled | Not possible | Not possible | Not possible | Not Possible |
| **Invasive Plant information** | Provided | Not provided | Not Provided | Not Provided | Not Provided |
| **Stress related Data** | Provided | Not Provided | Not Provided | Not Provided | Not Provided |
| **Chemical structure/formula** | Provided | Provided for few | Not Provided | Not Provided | Provided for few |
| **Identification Method** | Provided | Not Provided | Not Provided | Not Provided | Provided in some case |
| **Extraction method** | Provided for comparison | Not Provided | Provided | Not Provided | Provided |
| **Chemical category** | Provided | Not Provided | Not Provided | Not Provided | Provided |
| **Plant source organ** | Provided | Not Provided | Provided | Provided | Not Provided |
| **Biological Activity** | Provided | Not Provided | Not Provided | Provided | Not provided |
| **CAS number** | Provided | Provided | Not Provided | Not Provided | Not provided |
